# Long noncoding RNA lnc-DILC stabilizes PTEN and suppresses clear cell renal cell carcinoma progression

**DOI:** 10.1186/s13578-019-0345-4

**Published:** 2019-10-02

**Authors:** Han Zhang, Pengtao Wei, Wenwei Lv, Xingtao Han, Jinhui Yang, Shuaifeng Qin

**Affiliations:** Urology Department, Luoyang Central Hospital, No. 288, Zhongzhou Road, Luoyang, 471000 Henan China

**Keywords:** Ubiquitination, PTEN, WWP2, USP11

## Abstract

**Background:**

Increasing evidence has indicated that long noncoding RNAs (lncRNAs) are crucial regulators affecting the progression of human cancers. Recently, lncRNA downregulated in liver cancer stem cells (lnc-DILC) was identified to function as a tumor suppressor inhibiting the tumorigenesis and metastasis in liver cancer and colorectal cancer. However, to date, little is known about the functional roles of lnc-DILC in modulating malignant phenotypes of clear cell renal cell carcinoma (ccRCC) cells.

**Methods:**

lnc-DILC expression in human ccRCC tissues was detected by qRT-PCR. Overexpression and knockdown experiments were carried out to determine the effects of lnc-DILC on ccRCC cell proliferation, migration and invasion. To reveal the underlying mechanisms of lnc-DILC functions in ccRCC cells. RNA immunoprecipitation, RNA pull-down, in vivo ubiquitination, co-immunoprecipitation and western blot assays were performed.

**Results:**

Here, we identified that lnc-DILC levels were dramatically downregulated in ccRCC tissues. Loss of lnc-DILC expression was correlated with larger tumor size, advanced tumor grade and lymph node metastasis, and also predicted worse prognosis in patients with ccRCC. Functionally, knockdown and overexpression experiments demonstrated that lnc-DILC inhibited cell proliferation, migration and invasion in ccRCC cells. Mechanistic investigation revealed that lnc-DILC bound to tumor suppressor PTEN and suppressed its degradation. lnc-DILC repressed the PTEN ubiquitination through blocking the interaction between PTEN and E3 ubiquitin ligase WWP2 and recruiting the deubiquitinase USP11 to PTEN. Moreover, we demonstrated that PTEN–AKT signaling was crucial for lnc-DILC-mediated suppressive effects.

**Conclusions:**

In summary, our research revealed a novel mechanism by which lnc-DILC regulates PTEN stability via WWP2 and USP11, and shed light on potential therapeutic strategies by the restoration of lnc-DILC expression in patients with ccRCC.

## Background

Renal cell carcinoma (RCC) originates from renal tubular epithelial cells and is one of the most frequent cancers of the urinary system [1]. RCC can be divided into four subtypes, including chromophobe RCC, renal oncocytoma, clear cell RCC, and papillary RCC. Among them, clear cell RCC (ccRCC) is the most common subtype, and accounts for more than 70% of all RCC cases [2]. Patients with ccRCC are frequently not sensitive to radiotherapy and chemotherapy [3]. Since localized and distant metastasis or recurrence after surgical resection occurs in approximately 1/3 of patients, the prognosis of ccRCC patients remains unsatisfied [4]. Therefore, gaining insight into the underlying mechanisms of ccRCC progression will be helpful for finding the novel diagnosis and treatment for ccRCC.

Long noncoding RNAs (lncRNAs) are a group of transcripts more than 200 nucleotides in length and not capable to translated into proteins. LncRNAs were previously considered as “transcriptional junk” [5]. However, mounting evidence indicated that lncRNAs exert crucial regulatory functions. Dysregulation of some lncRNAs are closely associated with the initiation and progression of human cancers, such as liver cancer, lung cancer, breast cancer and ccRCC [6, 7]. LncRNAs play important roles in biological behavior of cancer cells, including cell proliferation, apoptosis, migration, invasion, autophagy, metabolism, senescence, differentiation and pluripotency [8–10]. LncRNAs exert their regulatory function via association with other molecules, such as mRNAs, microRNAs and proteins. For example, lncRNA HCAL associates with miR-196a/b and blocks miR-196a/b-mediated LAPTM4B suppression, which enhances growth and metastasis in liver cancer [11]. LncRNA CASC11 promotes osteosarcoma metastasis via directly interacting with snail mRNA and increasing its stability [12]. LINC00675 suppresses gastric cancer metastasis via increasing the phosphorylation of vimentin [13]. FAL1 associates with the epigenetic repressor BMI1 and stabilizes BMI1 protein to modulate the CDKN1A expression and tumor growth [14]. Several oncogenic lncRNAs have been characterized in ccRCC to date, including MALAT1 [15], PVT1 [16], URRCC [17], SNHG14 [18], lncARSR [19] and MRCCAT1 [20]. However, only a few tumor-suppressive lncRNAs and the exact mechanisms have been well investigated.

Lnc-DILC (lncRNA downregulated in liver cancer stem cells), a newly identified lncRNA, locates at the chromosomal locus 13p34. In liver cancer and colorectal cancer, lnc-DILC acts as a tumor suppressor to inhibit the tumorigenesis and metastasis. Lnc-DILC was found to suppress the IL-6/STAT3 signaling via inactivating IL-6 transcription [21, 22]. Conversely, lncDILC is upregulated in gallbladder carcinoma and facilitates the tumorigenicity and metastasis of gallbladder cancer cells via activating Wnt/β-catenin pathway [23]. However, whether lnc-DILC affects malignant behavior of ccRCC cells and the underlying mechanisms remain largely unstudied. Our present study aims to investigate the expression pattern, clinical significance, functional roles and molecular mechanisms of lnc-DILC in ccRCC progression. The results elucidates that lnc-DILC is a PTEN-interacting lncRNA, which stabilizes PTEN protein, thus inhibiting the development of ccRCC.

## Materials and methods

### Tissues collection

68 pairs of ccRCC and matched normal tissue samples were collected from ccRCC patients who underwent surgery at the Luoyang Central Hospital. None of patients received other therapy before surgery, such as chemotherapy, radiotherapy and targeted therapy. Written consent was obtained from all patients. This study was approved by the Institutional Ethics Committee of the Luoyang Central Hospital.

### Cell culture and transfection

The human ccRCC cell lines, ACHN, 786-O, 769-P, Caki-1 and OS-RC-2, and a normal human renal cell line HK-2 were purchased from the Cell Resources Center of Chinese Academy of Sciences. All cells were cultured in RPMI-1640 medium (Gibco) containing 10% FBS (Gibco) at 37 °C. siRNAs targeting lnc-DILC or PTEN was purchased from Ribobio Company (Guangzhou, China). Full-length lnc-DILC or PTEN was synthesized by GENECHEM Company (Shanghai, China) and subcloned into the pcDNA3.1 vector. Above siRNAs or plasmid were transfected into ccRCC cells by using Lipofectamine 2000 (Invitrogen) according to the the manufacturer’s instructions. 48 h after transfection, the cells were subjected to further experiments.

### qRT-PCR

Total RNA from tissue samples and cells were isolated by Trizol reagent (Invitrogen). The cDNA was synthesized by using EasyScript First-Strand cDNA Synthesis SuperMix Kit, and then detected by qRT-PCR using TransStart Green qPCR SuperMix (Transgen Biotech, China). The relative expression of indicated genes was normalized to GAPDH mRNA level and calculated using 2^−△△Ct^ methods. The primer sequences were shown as follows: lnc-DILC-sense: TGGCCTACTCCCAAGAAGATA, lnc-DILC-antisense: CATCCACAGCACGTCCTAAT; PTEN-sense: CCCACCACAGCTAGAACTTATC, PTEN-antisense: TCGTCCCTTTCCAGCTTTAC; GAPDH-sense: GGTGTGAACCATGAGAAGTATGA, GAPDH-antisense: GAGTCCTTCCACGATACCAAAG.

### Cell proliferation detection

Cell proliferation was assessed by CCK-8 (Dojindo) and clone formation assay according to the manufacturer’s instructions.

### Flow cytometry analysis

The cell apoptosis was detected using Annexin V-FITC Apoptosis Detection Kit (Sigma) according to the manufacturer’s instructions. For cell cycle analysis, cells were collected and fixed in 70% ethanol at 4 °C overnight and then incubated witi PI staining solution. Data were analyzed on a flow cytometer.

### Migration and invasion assay

Cell migration and invasion was examined with Transwell chambers (Corning) that coated without or with Matrigel (BD Biosciences), respectively. 1 × 10^5^ cells suspended in 200 μl serum-free RPMI-1640 medium were plated in the upper chambers, while 500 μl RPMI-1640 medium supplemented with 10% FBS was added to the bottom chamber. 24 h later, the cells on the lower surface were fixed, stained and counted.

### Western blot

Protein samples from cells were extracted by RIPA buffer (Beyotime, Beijing) and separated in SDS-PAGE gels and then transferred to PVDF membranes (Millipore). The membranes were incubated with primary antibodies overnight and then incubated with corresponding secondary antibodies. Protein expression was measured by enhanced chemiluminescence (Millipore).

### Caspase-3 activity detection

Caspase-3 activity was detected using the Caspase-3 activity assay kit (Cell signaling) according to the manufacturer’s instructions.

### Immunoprecipitation assay

Cell lysates were incubated with 3 μg PTEN antibody (Abcam) and Dynabeads Protein G (Invitrogen) on the rotating platform at 4 °C overnight. After wash, the immunoprecipitated protein was subjected to western blot analysis.

### In vivo ubiquitination assay

To test the ubiquitination level of PTEN, cells were pre-treated with 10 mM MG132 for 8 h. Cell lysates were incubated with 3 μg PTEN antibody (Abcam) and Dynabeads Protein G (Invitrogen) on the rotating platform at 4 °C overnight. After wash, the immunoprecipitated protein was subjected to western blot analysis using anti-Ubiquitin antibody (Cell signaling).

### RNA immunoprecipitation (RIP) and RNA pull-down assay

RIP and RNA pull-down assay was carried out using EZ-Magna RIP RNA-Binding Protein Immunoprecipitation Kit (Millopore) and Pierce Magnetic RNA–Protein Pull-Down Kit (Thermo Fisher) according to the the manufacturer’s instructions, respectively.

### Statistical analysis

All experiments were repeated at least three times. All statistical analyses were performed using SPSS 19.0. Student t-test or one way ANOVA test was used to assess the differences between groups. The correlation between lnc-DILC expression and clinicopathological parameters was analyzed by Chi-square test. The Kaplan–Meier method and log-rank test was used to evaluate the relationship between lnc-DILC expression and overall survival time. p value less than 0.05 were considered significant.

## Results

### Downregulation of lnc-DILC predicts poor clinical outcomes of patients with ccRCC

**Fig. 1:**
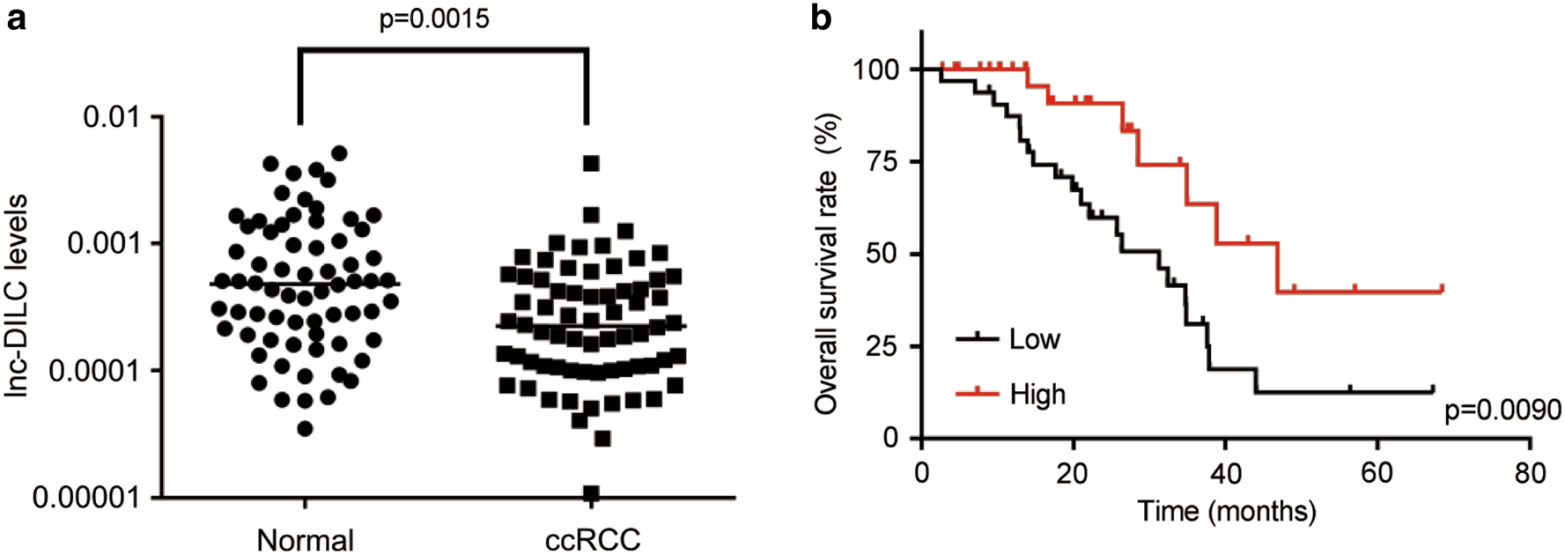
Downregulation of lnc-DILC expression predicts worse prognosis of patients with ccRCC. **a** The qRT-PCR assay was used to test the differential expression of lnc-DILC in 68 pairs of ccRCC and matched normal tissues. **b** The Kaplan–Meier method and log-rank test was used to evaluate the relationship between lnc-DILC expression and overall survival time of ccRCC patients

We first determined the expression pattern of lnc-DILC in ccRCC. The qRT-PCR assay was carried out to examine the lnc-DILC levels in 68 pairs of ccRCC and matched normal tissue samples. As shown in Fig. 1a, lnc-DILC expression was remarkably decreased in ccRCC tissues than normal tissues. To investigate the clinical significance of aberrant expression of lnc-DILC, the patients were divided into high-expression and low-expression groups according to the median value of lnc-DILC expression in ccRCC tissues. Correlation analysis between lnc-DILC expression and clinicopathological features showed that decrease of lnc-DILC expression associated with larger tumor size, advanced Fuhrman tumor grade and lymph node metastasis (Table 1). Nevertheless, no significant correlation between lnc-DILC expression and other features was observed, including age, gender and smoke status. Furthermore, we evaluated the prognostic value of lnc-DILC expression in ccRCC patients through Kaplan–Meier method, and the results demonstrated high-expression group had a better overall survival, as compared to low-expression group (Fig. 1b). These results indicate that lnc-DILC may function as a tumor suppressor in ccRCC, and loss of lnc-DILC expression may contribute to ccRCC progression.

**Table 1:** The correlation between the expression of lnc-DILC and clinicopathological features of patients with ccRCC

Variables	Expression of lnc-DILC		*p*-value
	Low	High	
Gender			0.417
Male	26	23	
Female	8	11	
Age			0.801
≤ 60	21	22	
> 60	13	12	
Smoke			0.806
Yes	15	14	
No	19	20	
Fuhrman grade			0.001
G1–G2	27	14	
G3–G4	7	20	
Lymph node metastasis			0.029
Yes	22	13	
No	12	21	
Tumor size (cm)			0.003
≤ 7	26	14	
> 7	8	20	

### lnc-DILC suppresses cell proliferation in ccRCC cells

**Fig. 2:**
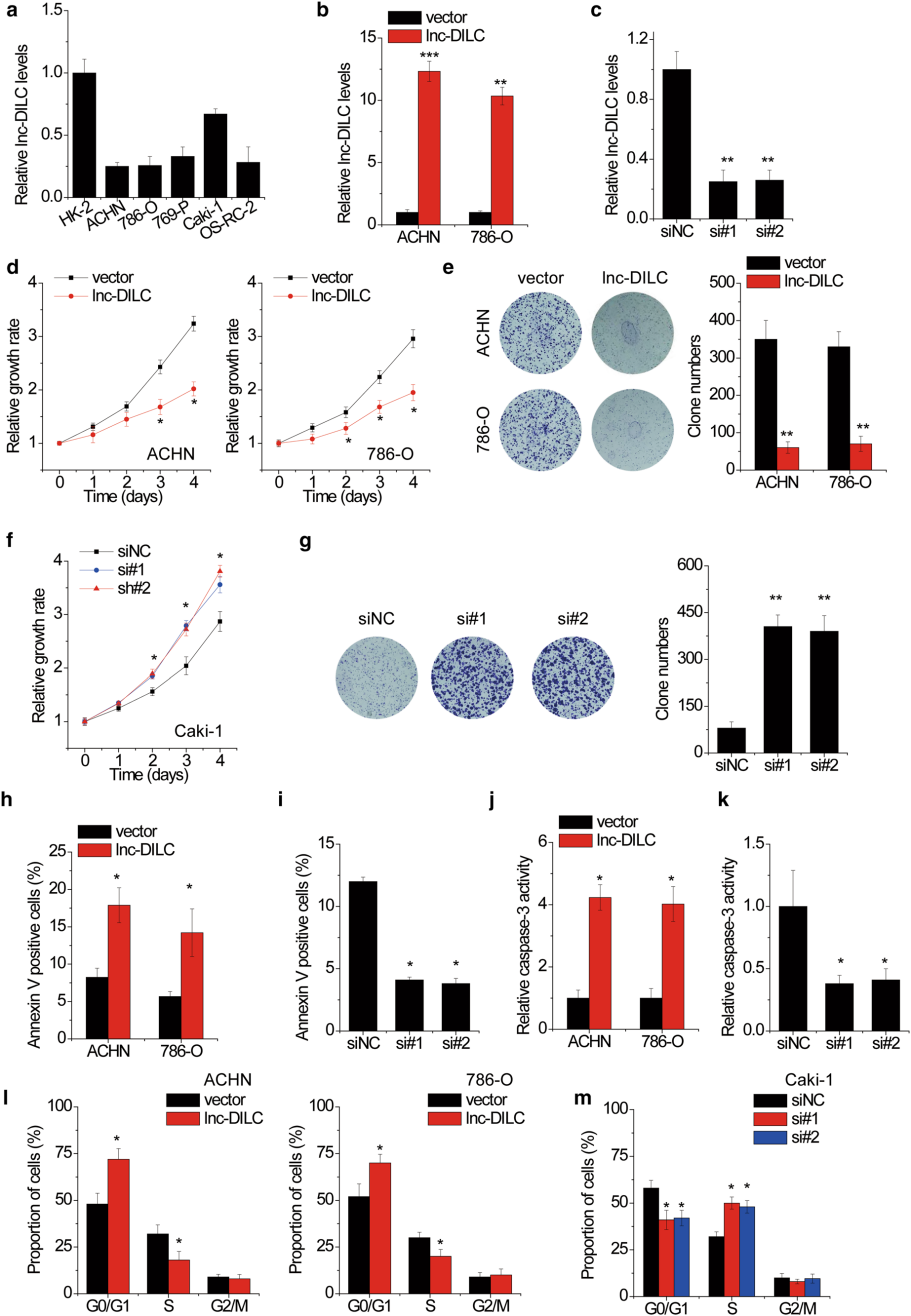
lnc-DILC reduces the proliferative ability of ccRCC cells. **a** lnc-DILC expression levels in the ccRCC cell lines and a normal human renal cell line (HK-2) were detected by qRT-PCR. **b** qRT-PCR assays for the lnc-DILC levels in ACHN and 786-O cells transfected with pcDNC3.1-lnc-DILC or the empty vector. **c** qRT-PCR assays for the lnc-DILC levels in Caki-1 cells transfected with two different siRNAs targeting lnc-DILC (si#1 and si#2). **d** The cell proliferation of ACHN and 786-O in response to lnc-DILC knockdown was measured using CCK-8 assay. **e** Colony formation assays performed with the ACHN and 786-O cells transfected with pcDNC3.1-lnc-DILC or the empty vector. **f** The cell proliferation of Caki-1 in response to lnc-DILC overexpression was measured using CCK-8 assay. **g** Colony formation assays performed with the Caki-1 cells transfected with lnc-DILC or negative control siRNAs. **h** Apoptosis was assayed by flow cytometry in ACHN and 786-O cells after overexpression of lnc-DILC. **i** Apoptosis was assayed by flow cytometry in Caki-1 cells after knockdown of lnc-DILC. **j** Caspase-3 activity of ACHN and 786-O cells was assayed after overexpression of lnc-DILC. **k** Caspase-3 activity of Caki-1 cells was assayed after knockdown of lnc-DILC. **l** The cell cycle analysis of ACHN and 786-O transfected with pcDNC3.1-lnc-DILC or the empty vector was performed using flow cytometry. The cell cycle distribution was listed with the percentage of G1, S, and G2 phases in the graphs. **m** The cell cycle analysis of Caki-1 cells transfected with lnc-DILC or negative control siRNAs was performed using flow cytometry. *p < 0.05, **p < 0.01, ***p < 0.001

To explore the biological function of lnc-DILC in ccRCC progression, the lnc-DILC expression in five ccRCC cell lines (ACHN, 786-O, 769-P, Caki-1 and OS-RC-2) was assessed using qRT-PCR assay (Fig. 2a). According to the endogenous lnc-DILC expression level, pcDNA3.1-lnc-DILC was transfected into the ACHN and 786-O cell line with relatively low expression of lncDILC, while two independent siRNAs targeting lnc-DILC were transfected into the Caki-1 cell line with relatively high expression of lncDILC. The efficiency of knockdown and overexpression of lnc-DILC expression was validated by qRT-PCR (Fig. 2b, c). CCK-8 and clone formation assays were carried out to examine the effect of lnc-DILC alteration on ccRCC cell proliferation. It was demonstrated that the proliferative capacity of ACHN and 786-O cells was significantly repressed by lnc-DILC overexpression (Fig. 2d, e), whereas depletion of lnc-DILC expression enhanced the cell proliferation in Caki-1 cells (Fig. 2f, g).

We next assessed the influence of lnc-DILC alteration on cell apoptosis and cell cycle distribution in ccRCC. The flow cytometry analysis showed that overexpression of lnc-DILC markedly increased the apoptotic rate of ACHN and 786-O cells (Fig. 2h), while silence of lnc-DILC showed a protective effect in Caki-1 cells (Fig. 2i). For further confirmation, fluorometric enzyme assay was utilized to detect the caspase-3 activity, and the results showed that activity of caspase-3 were significantly enhanced by ectopic expression of lnc-DILC in ACHN and 786-O cells (Fig. 2j), but decreased by transfection of lnc-DILC siRNAs in Caki-1 cells (Fig. 2k). The cell cycle analysis via low cytometry assay demonstrated an increased percentage of G0/G1 phase and a decreased percentage of S phase in lnc-DILC-overexpressed ACHN and 786-O cells compared with the control groups (Fig. 2l). Conversely, the depletion of endogenous lnc-DILC induced the progression of G1-to-S phase transformation (Fig. 2m).

### lnc-DILC inhibits cell migration and invasion in ccRCC

**Fig. 3:**
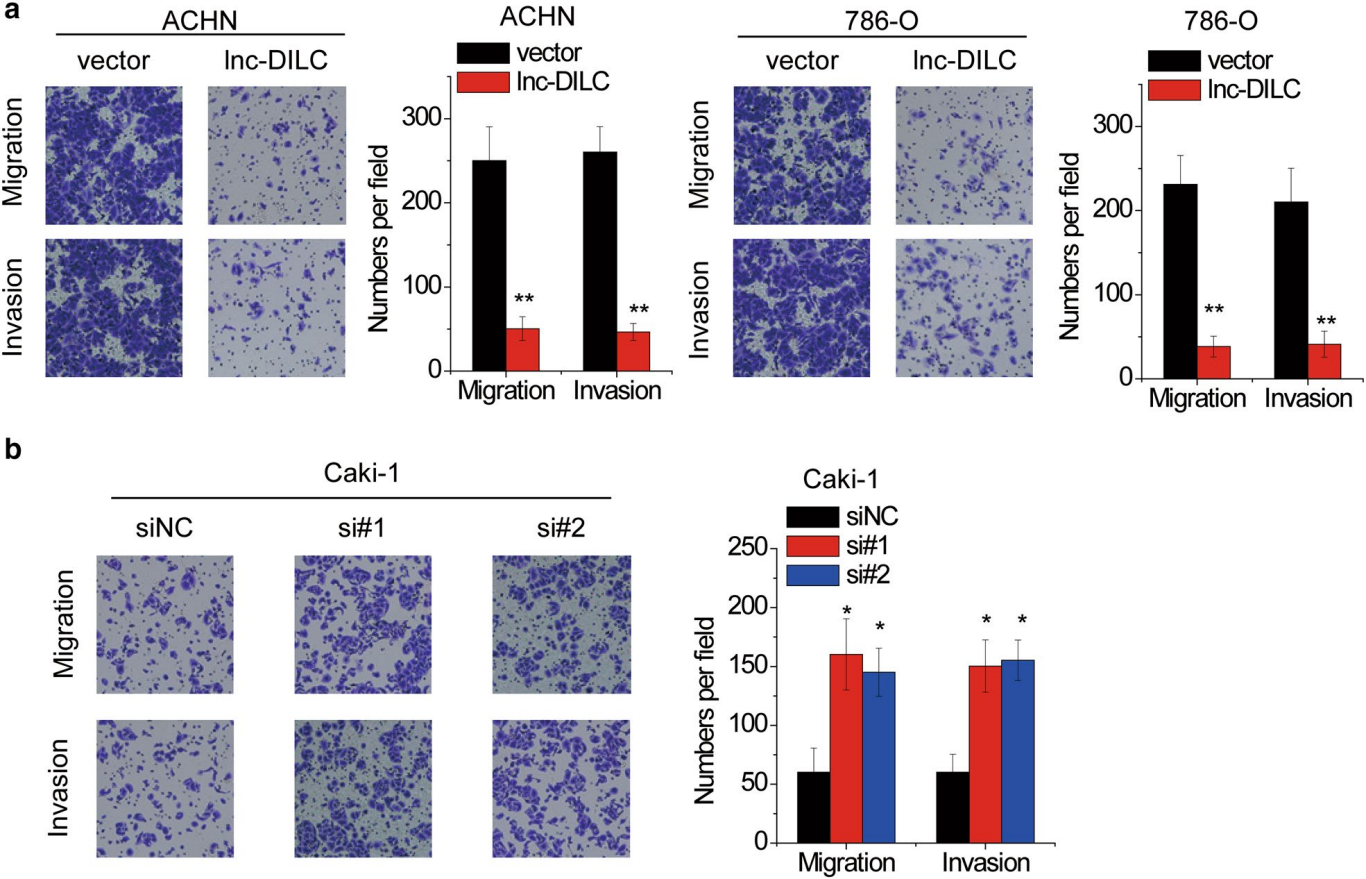
lnc-DILC suppresses migration and invasion in ccRCC cells. **a** Representative images of ACHN and 786-O cell migration or invasion across the membrane after lnc-DILC overexpression. The histograms show the average number of migrated or invasive cells per field calculated from five representative fields. **b** Representative images of Caki-1 cell migration or invasion across the membrane after lnc-DILC knockdown. The histograms show the average number of migrated or invasive cells per field calculated from five representative fields. *p < 0.05, **p < 0.01

Given the significant correlation between lnc-DILC expression and lymph node metastasis (Table 1), we speculated that lnc-DILC contribute to the cell migration and invasion in ccRCC cells. Transwell migration and invasion assay demonstrated that the migratory and invasive capability of ACHN and 786-O cells was markedly attenuated after transfection of lnc-DILC (Fig. 3a). Reciprocally, knockdown of lnc-DILC significantly facilitated the Caki-1 cells migration and invasion compared to the control group (Fig. 3b).

### lnc-DILC associates with PTEN and increases its stability

**Fig. 4:**
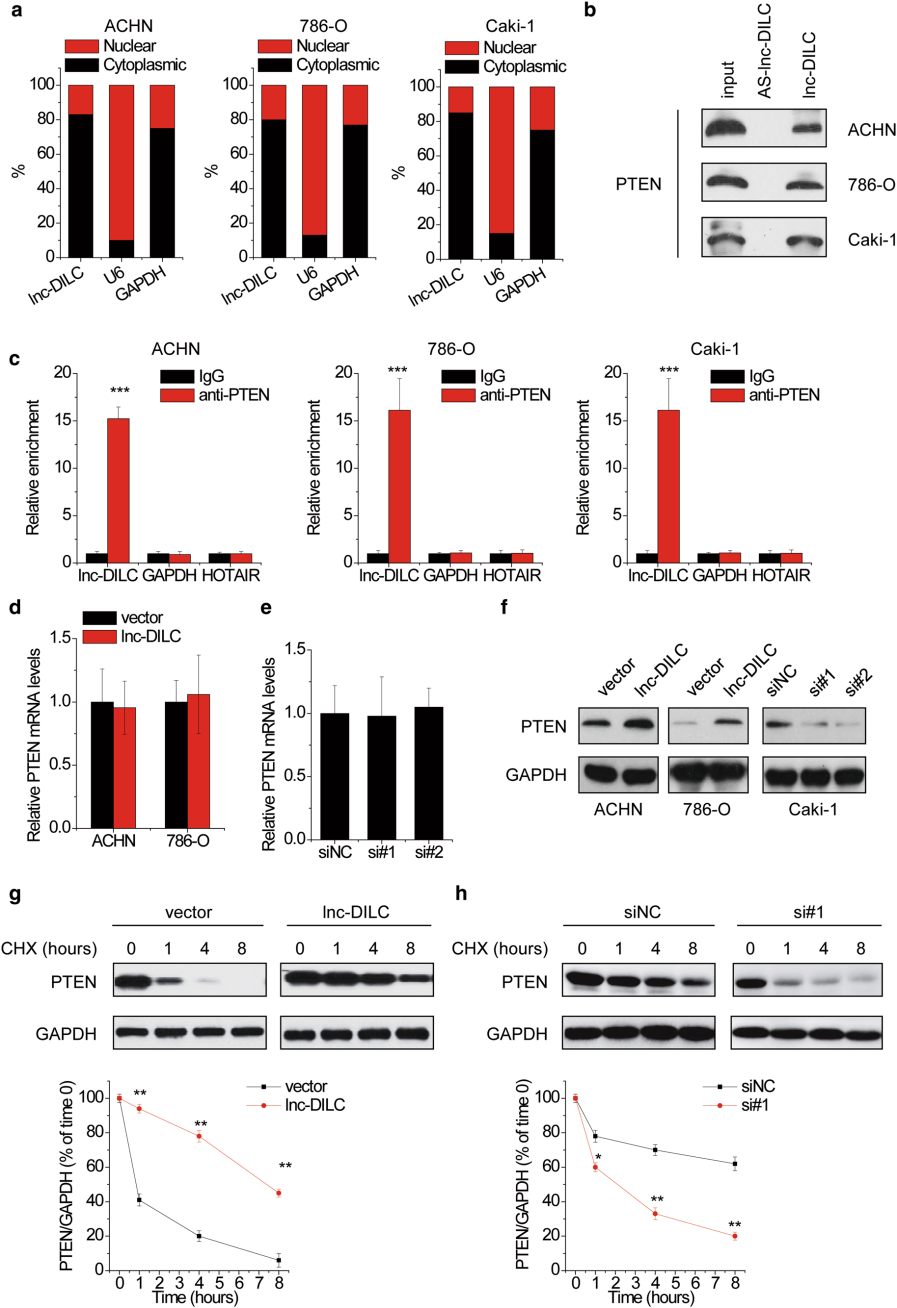
lnc-DILC suppresses PTEN degradation. **a** The subcellular location of lnc-DILC in ccRCC cells. U6 and GAPDH were used as internal controls. **b** Biotinylated lnc-DILC or antisense lnc-DILC (AS-lnc-DILC) were incubated with ccRCC cell extracts, targeted with streptavidin beads, and associated proteins were resolved in a gel. Western blotting analysis of the specific association of PTEN and lnc-DILC. Antisense lncDILC was shown as a negative control. **c** RIP experiments were performed to examine the RNA associated with PTEN. HOTAIR and GAPDH mRNA was taken as negative control. **d** The PTEN mRNA levels in ACHN and 786-O cells transfected with pcDNC3.1-lnc-DILC or the empty vector. **e** The PTEN mRNA levels in Caki-1 cells transfected with lnc-DILC or negative control siRNAs. **f** The PTEN protein levels in ccRCC cells with lnc-DILC overexpression or knockdown were determined by western blot. **g** ACHN transfected with pcDNC3.1-lnc-DILC or the empty vector were treated with CHX (0.1 mg/ml) and harvested at the indicated times. Cells were lysed and cell lysates were then blotted with the indicated antibodies. Lower panel: quantification of the PTEN protein levels relative to GAPDH. **h** Caki-1 cells transfected with lnc-DILC or negative control siRNAs were treated with CHX (0.1 mg/ml) and harvested at the indicated times. Cells were lysed and cell lysates were then blotted with the indicated antibodies. Lower panel: quantification of the PTEN protein levels relative to GAPDH. *p < 0.05, **p < 0.01, ***p < 0.001

To investigate the underlying mechanism of the effect of lnc-DILC on malignant phenotypes of ccRCC, we examined the subcellular location of lnc-DILC in ccRCC cells. The lnc-DILC expression in separated cytoplasm RNA and nuclear RNA was analyzed. It was observed that lnc-DILC was preferentially located in the cytoplasm (Fig. 4a), indicating that lnc-DILC may be involved in post-transcriptional or post-translational regulation. We then performed RNA pull-down assay and followed by mass spectrum analysis to identify the candidate proteins which interacted with lnc-DILC (Additional file 1: Figure S1A). Interestingly, PTEN, a well-known tumor suppressor, was found to potentially associate with lnc-DILC. To validate this result, RNA pull-down assay was conducted, and the results indicated that endogenous PTEN protein could be specifically pulled down by lnc-DILC, but not the antisense lnc-DILC, which was used as negative controls (Fig. 4b). For further confirmation, RIP assay was carried out, and the results showed that endogenous lnc-DILC instead of HOTAIR or GAPDH mRNA could be significantly enriched by PTEN antibody compared to negative control IgG (Fig. 4c).

Next, the regulatory relationship between lnc-DILC and PTEN was investigated. Neither knockdown nor overexpression of lnc-DILC affected PTEN mRNA levels (Fig. 4d, e). Notably, PTEN protein level was significantly increased after lnc-DILC overexpression in ACHN and 786-O cells. In contrast, transfection of lnc-DILC siRNAs led to a markedly decrease of PTEN protein level in Caki-1 cells (Fig. 4f). PTEN protein level was also positively correlated with lnc-DILC expression level in ccRCC cell lines (Additional file 1: Figure S1B). These results indicated that lnc-DILC regulated PTEN expression in a posttranslational manner. Additionally, cells with lnc-DILC alteration were treated with CHX (protein synthesis inhibitor) to examine whether lnc-DILC affected the half-life of PTEN protein. It was observed that overexpression of lnc-DILC prolonged the half-life of PTEN in ACHN cells (Fig. 4g), whereas knockdown of lnc-DILC exerted opposite effects in Caki-1 cells (Fig. 4h). Collectively, our findings suggested that lnc-DILC suppresses the degradation of PTEN.

### lnc-DILC impedes PTEN ubiquitination through preventing the interaction between PTEN and E3 ubiquitin ligase WWP2

**Fig. 5:**
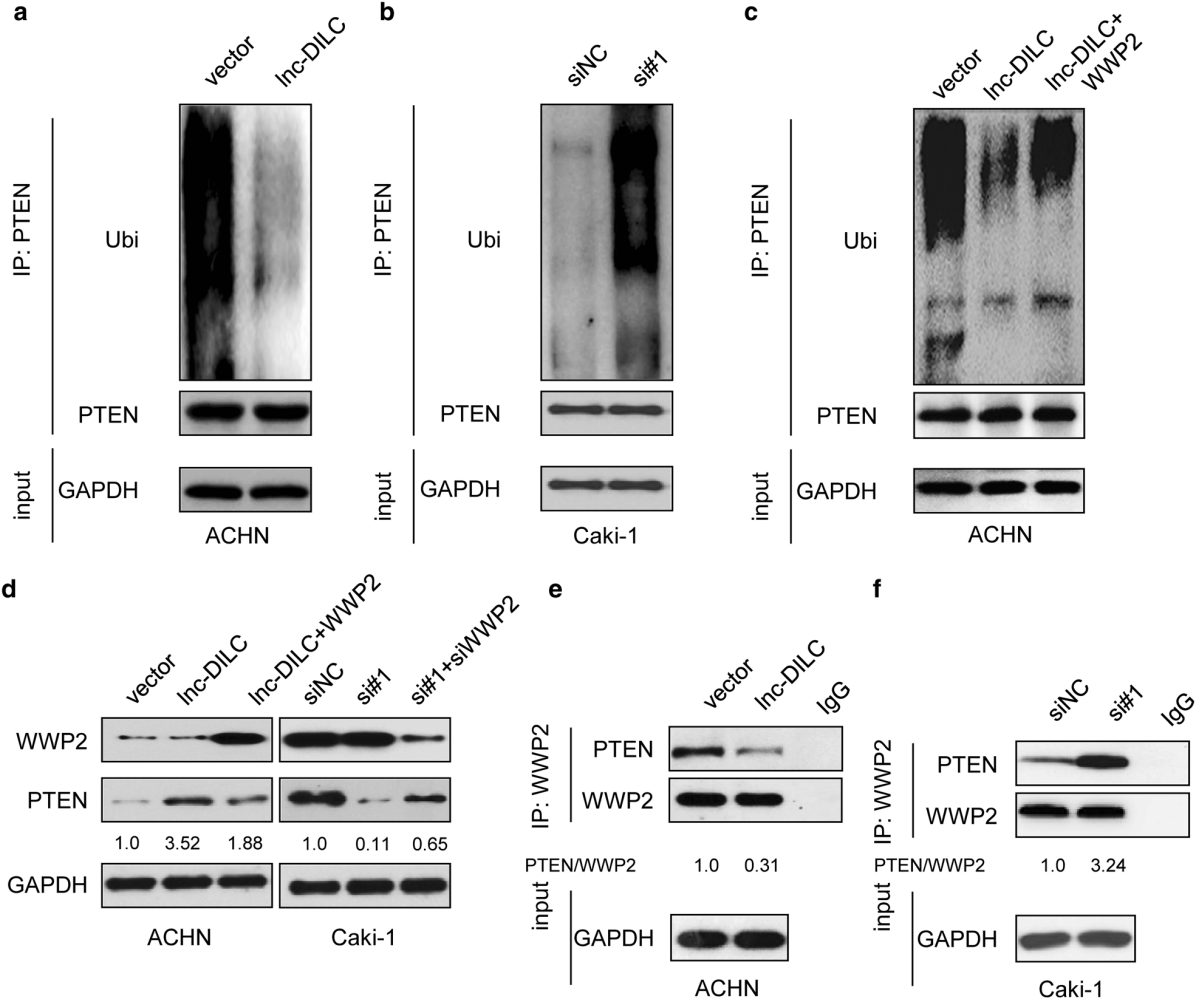
lnc-DILC inhibits the interaction between PTEN and WWP2. **a** ACHN cells transfected with pcDNC3.1-lnc-DILC or the empty vector were treated with MG132 for 4 h before harvest. PTEN was immunoprecipitated and immunoblotted with the indicated antibodies. **b** Caki-1 cells transfected with lnc-DILC or negative control siRNAs were treated with MG132 for 4 h before harvest. PTEN was immunoprecipitated and immunoblotted with the indicated antibodies. **c** ACHN cells cotransfected with pcDNC3.1-lnc-DILC and pcDNA3.1-WWP2 were treated with MG132 for 4 h before harvest. PTEN was immunoprecipitated and immunoblotted with the indicated antibodies. **d** ACHN and Caki-1 cells with lnc-DILC alteration were cotransfected with pcDNA3.1-WWP2 and WWP2 siRNAs, respectively. 48 h later, the PTEN was detected by western blot assay. **e** The effect of lnc-DILC overexpression on the interaction between PTEN and WWP2 was determined by co-immunoprecipitation assay in ACHN cells. **f** The effect of lnc-DILC knockdown on the interaction between PTEN and WWP2 was determined by co-immunoprecipitation assay in Caki-1 cells

Ubiquitination modification plays an important role in PTEN degradation. We carried out an ubiquitination assay to determine whether lnc-DILC attenuated the ubiquitination of PTEN, and the results demonstrated that ectopic expressed lnc-DILC significantly decreased the ubiquitination level of PTEN in ACHN cells (Fig. 5a). Conversely, knockdown of lnc-DILC increased PTEN ubiquitination in Caki-1 cells (Fig. 5b). Previous studies have reported that WWP2 is an E3 ubiquitin ligase which can specially bind to PTEN and induces its degradation [24, 25]. Interestingly, we found that ectopic expression of WWP2 could partially abolished the PTEN ubiquitination reduced by lnc-DILC (Fig. 5c). Moreover, WWP2 overexpression partly reversed the lnc-DILC-mediated PTEN upregulation in ACHN cells, whereas silence of WWP2 partially rescued the downregulation of PTEN mediated by lnc-DILC knockdown in Caki-1 cells (Fig. 5d). Furthermore, the co-immunoprecipitation was performed to determine whether lnc-DILC affected the interaction between PTEN and WWP2 and the results demonstrated that overexpression of lnc-DILC significantly attenuated the binging of WWP2 with PTEN in ACHN cells (Fig. 5e), whereas deletion of lnc-DILC1 enhanced the interaction between WWP2 and PTEN in Caki-1 cells (Fig. 5f). These results indicate that lnc-DILC suppressed the PTEN ubiquitination partially through suppressing the interaction between PTEN and WWP2.

### lnc-DILC also recruits deubiquitinase USP11 to stabilize PTEN

**Fig. 6:**
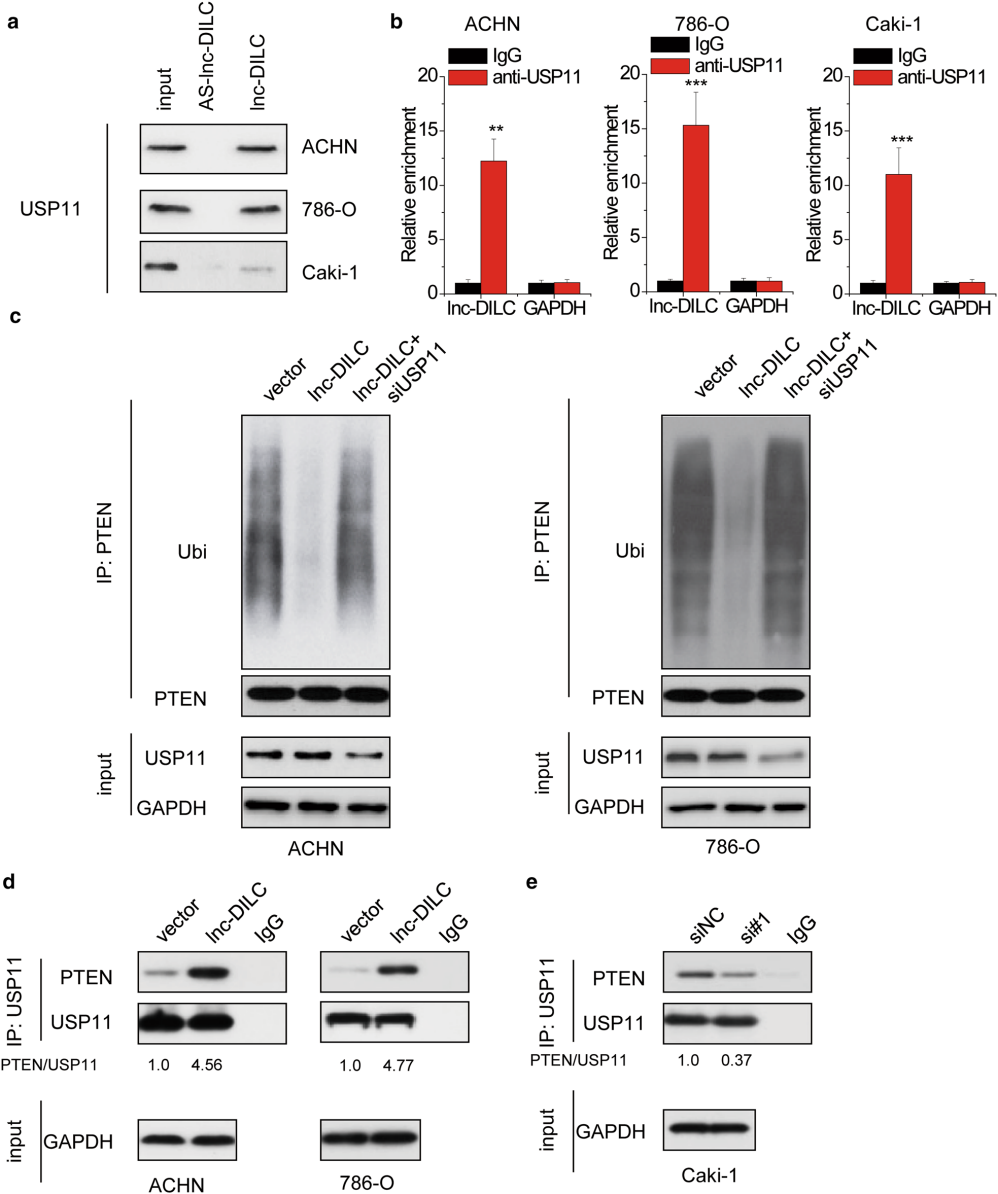
lnc-DILC enhances the interaction between PTEN and USP11. **a** Biotinylated lnc-DILC or antisense lnc-DILC (AS-lnc-DILC) were incubated with ccRCC cell extracts, targeted with streptavidin beads, and associated proteins were resolved in a gel. Western blotting analysis of the specific association of USP11 and lnc-DILC. Antisense lncDILC was shown as a negative control. **b** RIP experiments were performed to examine the RNA associated with USP11. GAPDH mRNA was taken as negative control. **c** ACHN and 786-O cells cotransfected with pcDNC3.1-lnc-DILC and USP11 siRNAs were treated with MG132 for 4 h before harvest. PTEN was immunoprecipitated and immunoblotted with the indicated antibodies. **d** The effect of lnc-DILC overexpression on the interaction between PTEN and USP11 was determined by co-immunoprecipitation assay in ACHN and 786-O cells. **e** The effect of lnc-DILC knockdown on the interaction between PTEN and USP11 was determined by co-immunoprecipitation assay in Caki-1 cells. **p < 0.01, ***p < 0.001

We then explored other mechanism of the effect of lnc-DILC on PTEN expression. Recently, deubiquiting enzyme USP11 has been found to regulate PTEN stability [26]. Notably, the results of RNA pull-down assay and followed by mass spectrum analysis showed that USP11 may potentially associated with lnc-DILC. The RNA pull-down and RIP assays validated an association between lnc-DILC and USP11 (Fig. 6a, b). Furthermore, depletion of USP11 by siRNAs attenuated the lnc-DILC-induced deubiquitination of PTEN (Fig. 6c). co-immunoprecipitation assays were performed to assess the influence of lnc-DILC overexpression or knockdown in the interaction of PTEN and USP11, and the results demonstrated a promotive effect of lnc-DILC on PTEN-USP11 association (Fig. 6d, e).

### AKT signaling pathway involves in the suppressive effects of lnc-DILC

**Fig. 7:**
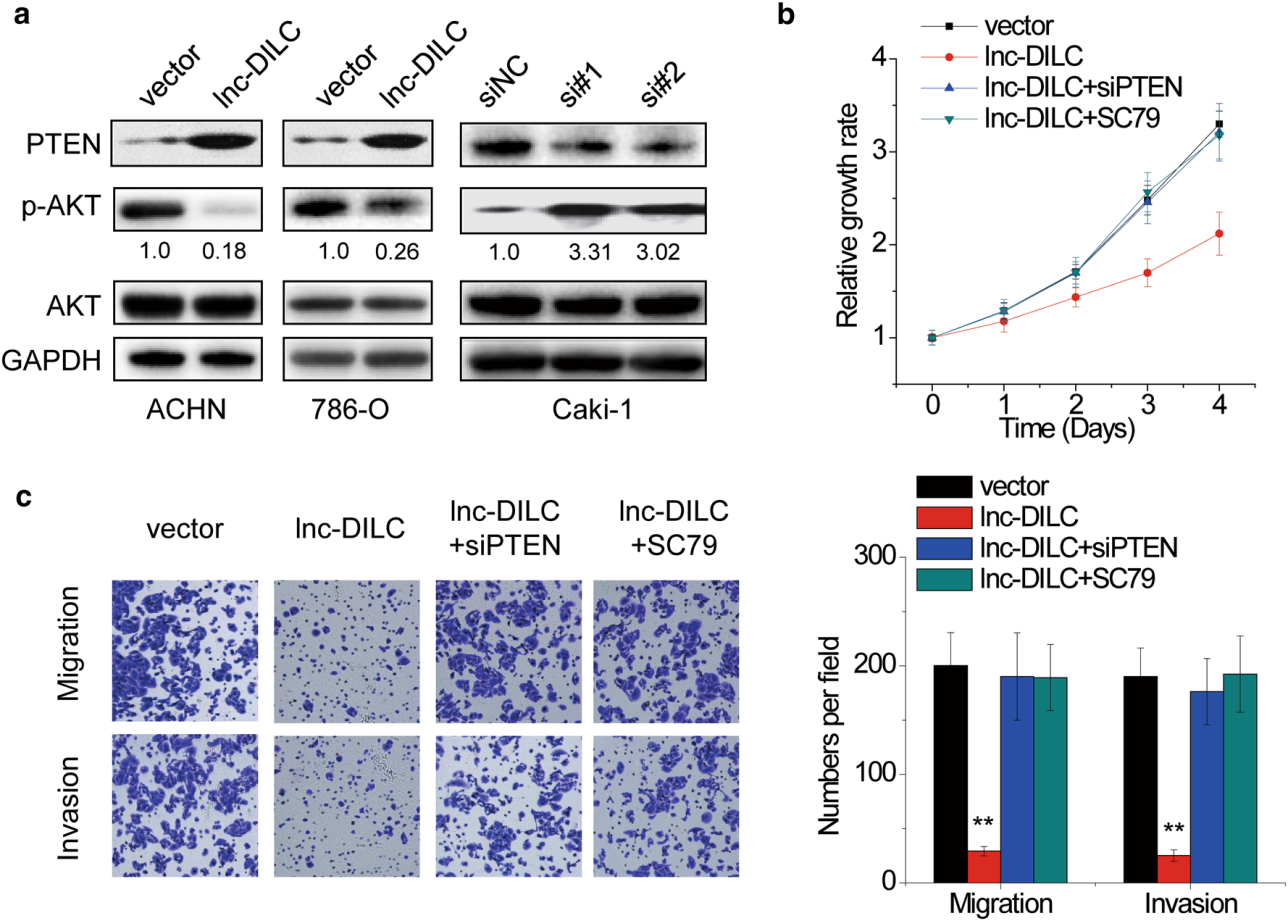
AKT signaling pathway involves in the suppressive effects of lnc-DILC. **a** The phosphorylation of AKT (p-AKT) was detected by western blot in ccRCC cells with lnc-DILC alteration. **b** The ACHN cells with lnc-DILC overexpression were transfected with PTEN siRNAs or treated with the pharmacological activator of AKT signaling (SC79), and then the cell proliferation was determined by CCK-8 assay. **c** The ACHN cells with lnc-DILC overexpression were transfected with PTEN siRNAs or treated with the pharmacological activator of AKT signaling (SC79), and then the cell migration and invasion was determined by Transwell assay. *p < 0.05, **p < 0.01, ***p < 0.001

AKT signaling is important for ccRCC progression and suppressed by PTEN [27, 28], indicating that lnc-DILC may negatively regulate the activation of AKT signaling via PTEN. To validate this, we performed western blot assay, and the results demonstrated that overexpression of lnc-DILC decreased the phosphorylation of AKT in ACHN and 786-O cells, while knockdown of lnc-DILC enhanced AKT phosphorylation in Caki-1 cells (Fig. 7a). Furthermore, rescue experiments identified that the phenotypes caused by lnc-DILC overexpression could be abolished by transfection of PTEN siRNAs or using the pharmacological activator of AKT signaling (SC79) in ACHN cells (Additional file 2: Figure S2, Fig. 7b, c). Taken together, our findings suggested that lnc-DILC inhibits ccRCC progression by PTEN–AKT signaling pathway.

## Discussion

Here, we identified lnc-DILC acting as a tumor suppressor lncRNA in ccRCC progression. Our research is the first to report the expression pattern, functional roles and underlying mechanism of lnc-DILC in regulating malignant phenotypes of ccRCC cells. We found that downregulation of lnc-DILC expression in ccRCC tissues correlated with larger tumor size, Fuhrman tumor grade, lymph node metastasis, and predicted shorter overall survival time of ccRCC patients (Table 1). Lnc-DILC inhibited cell proliferation, migration and invasion in ccRCC cells by stabilizing PTEN through two different manners which lnc-DILC interrupted the PTEN–WWP2 interaction and recruited USP11 to PTEN. All these data strongly suggested the importance of lnc-DILC in suppressing ccRCC progression, and that lnc-DILC could act as a candidate prognostic biomarker for ccRCC patients.

lncRNAs play important roles in tumor growth and metastasis via different mechanisms including *cis*- or *trans*-regulation, RNA scaffold, microRNA sponge, RNA decay, epigenetic modification, post-translational modification [7, 29, 30]. Aberrant post-translational modifications of protein contribute to ccRCC initiation and progression. For example, the phosphorylation and ubiquitination modification regulated the activity of serine/threonine protein phosphatase 5, which regulated cell apoptosis in ccRCC apoptosis [31]. Unsaturated free fatty disrupts the FAF1/β-catenin complex, decreases β-catenin ubiquitination and inhibits its degradation, which stimulates cell proliferation in ccRCC cells [32]. The cytoplasmic accumulation of SPOP induces the ubiquitination of PTEN, ERK phosphatases, Daxx and Gli2 to promote tumorigenesis in ccRCC [33]. However, the significance of lncRNAs in post-translational modification during ccRCC progression remains elusive. Here, we identified lnc-DILC as a PTEN-interacting lncRNA in ccRCC cells. lnc-DILC directly interacted with PTEN, suppressing its ubiquitination and degradation. Previous studies have demonstrated that protein ubiquitination could be regulated by aberrant expression of lncRNAs in human cancers, such as NBAT1 [34], lnc-UICC [35] and OCC-1 [36]. Combined with these researches, our present study further emphasized the importance of lncRNAs-mediated ubiquitination modification of their interacting proteins in regulating cancer progression.

PTEN is a well-known tumor suppressor inhibiting tumor growth and metastasis. In cytoplasm, PTEN depshophorylates AKT activator PIP3, subsequently inactivating AKT signaling [37]. Additionally, nuclear PTEN is capable to maintain genome stability [38]. Dysregulation of PTEN expression is mainly due to transcriptional and posttranslational modifications [39–41]. Recently, lncRNAs were found to take part in the regulation of PTEN through different mechanisms. Some lncRNAs function as competing endogenous RNA to sponge target microRNA and block its suppression on PTEN, such as lncRNA-ORLNC1 [42], TP73-AS1 [43] and HOTAIR [44]. However, little is known about the underlying mechanism by which lncRNAs regulated the stability of PTEN. lncRNA TTN-AS1 promotes the degradation of PTEN through inhibiting the association between PTEN and MAGI2 [45]. Linc02023 suppresses colorectal cancer growth through blocking the interaction between PTEN and WWP2 and enhancing PTEN stability [46]. Here, we demonstrated that lnc-DILC also stabilized PTEN through two different mechanisms. One hand, lnc-DILC disrupted the binding of WWP2 on PTEN, suppressing the WWP2-induced the ubiquitination of PTEN. We speculated that lnc-DILC may occupy the binding sites of WWP2 on PTEN, thus blocking the PTEN–WWP2 interaction, which needs further exploration. On the other hand, lnc-DILC recruited deubiquitinase USP11 to PTEN, thus reducing the ubiquitination level of PTEN. The function of USP11 in different types of cancers is paradoxical. In breast cancer, USP11 promotes the TGF-β-induced epithelial–mesenchymal transition via altering the stability of TGFβ receptor type II [47]. USP11-mediated deubiquitination of p21 inhibits non-small cell lung cancer growth in vivo [48]. PTEN activates the transcription of USP11 by the PI3K/FOXO pathway to enhance its own stability in prostate cancer cell line [26]. Nevertheless, USP11 functions as an oncogene in liver cancer to promote growth and metastasis [49]. Here, we showed that USP11 involved in lnc-DILC-mediated stabilization of PTEN, suggesting that USP11 may act as a tumor suppressor in ccRCC.

## Conclusion

Collectively, we revealed a new mechanism by which lnc-DILC enhances PTEN stability through affecting the interaction of PTEN with WWP2 and USP11. Our findings shed light on potential therapeutic strategies by the restoration of lnc-DILC expression in patients with ccRCC.

## Supplementary information

**Additional file 1: Figure S1.** lnc-DILC suppresses PTEN degradation. A. Biotin-RNA pull-downs were performed with extracts of cells using full-length lnc-DILC transcript. This was followed by mass spectrometry. B. The protein levels in the ccRCC cell lines were determined by western blot.

**Additional file 2: Figure S2.** AKT signaling pathway involves in the suppressive effects of lnc-DILC. Western blot of PTEN, p-AKT and cyclin D1 (p-AKT down-stream protein) levels when treated by PTEN siRNA or SC79 in lnc-DILC-overexpressed ACHN cell.

## Data Availability

The datasets used during this research are available.

## Abbreviations

lncRNAs: long noncoding RNAs
lnc-DILC: liver cancer stem cells
RCC: renal cell carcinoma
ccRCC: clear cell renal cell carcinoma
RIP: RNA immunoprecipitation
