# Supplementary figures and images for: Long noncoding RNA lnc-DILC stabilizes PTEN and suppresses clear cell renal cell carcinoma progression
Source: Cell Biosci. 2019 Oct 2;9:81. doi: 10.1186/s13578-019-0345-4 (PMC6775667; doi:10.1186/s13578-019-0345-4)

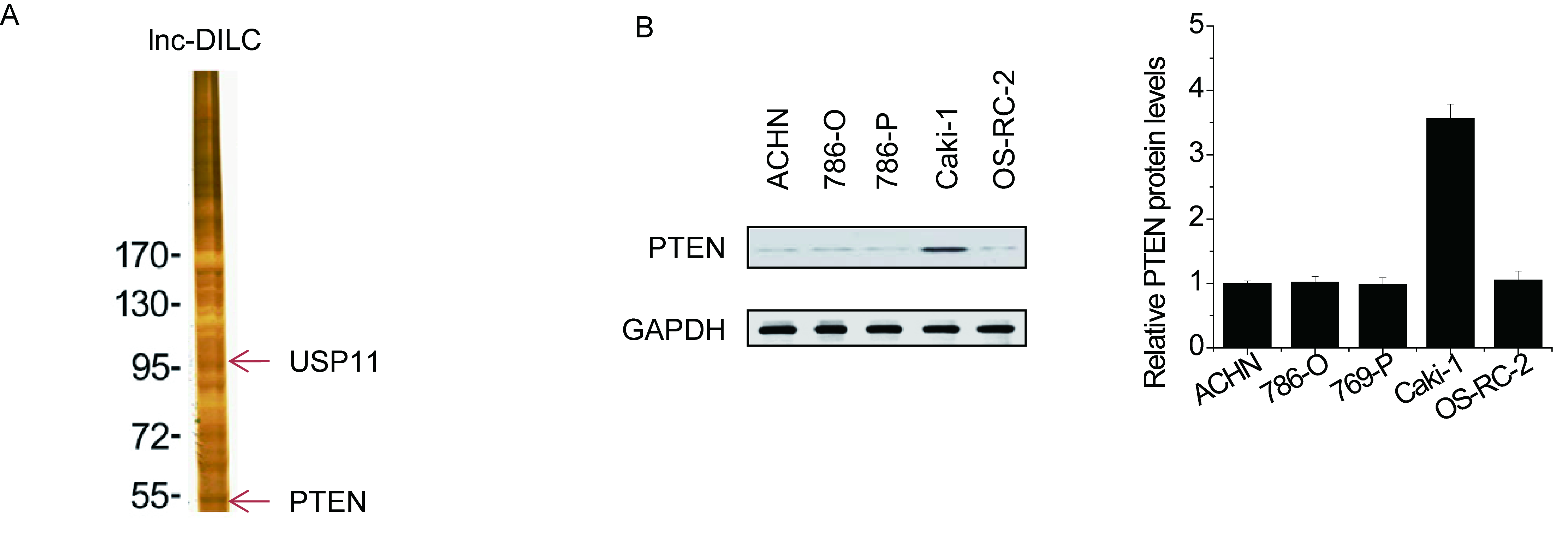

Supplement: Supplementary file 1 — Additional file 1: Figure S1. lnc-DILC suppresses PTEN degradation. A. Biotin-RNA pull-downs were performed with extracts of cells using full-length lnc-DILC transcript. This was followed by mass spectrometry. B. The protein levels in the ccRCC cell lines were determined by western blot. [file 13578_2019_345_MOESM1_ESM.tif]

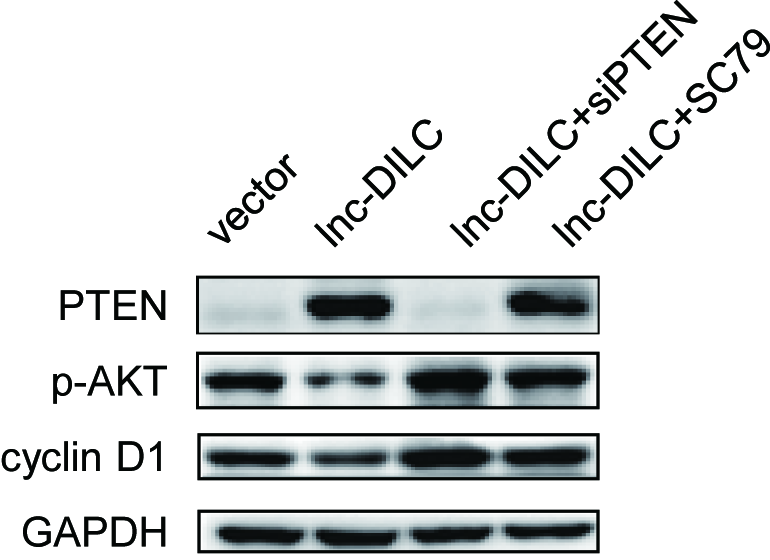

Supplement: Supplementary file 2 — Additional file 2: Figure S2. AKT signaling pathway involves in the suppressive effects of lnc-DILC. Western blot of PTEN, p-AKT and cyclin D1 (p-AKT down-stream protein) levels when treated by PTEN siRNA or SC79 in lnc-DILC-overexpressed ACHN cell. [file 13578_2019_345_MOESM2_ESM.tif]
